# Depression, mitochondrial bioenergetics, and electroconvulsive therapy: a new approach towards personalized medicine in psychiatric treatment - a short review and current perspective

**DOI:** 10.1038/s41398-020-00901-7

**Published:** 2020-07-09

**Authors:** Dr. Alexander Karabatsiakis, Prof. Dr. Carlos Schönfeldt-Lecuona

**Affiliations:** 1 Institute of Psychology, University of Innsbruck, Innsbruck, Tyrol Austria; 2 Clinic for Psychiatry and Psychotherapy III, Ulm University Clinic, Ulm, Baden-Wuerttemberg Germany

**Keywords:** Physiology, Diagnostic markers, Prognostic markers, Psychiatric disorders

## Abstract

Major depressive disorder (MDD) is a globally occurring phenomenon and developed into a severe socio-economic challenge. Despite decades of research, the underlying pathophysiological processes of MDD remain incompletely resolved. Like other mental disorders, MDD is hypothesized to mainly affect the central nervous system (CNS). An increasing body of research indicates MDD to also change somatic functioning, which impairs the physiological performance of the whole organism. As a consequence, a paradigm shift seems reasonable towards a systemic view of how MDD affects the body. The same applies to treatment strategies, which mainly focus on the CNS. One new approach highlights changes in the bioenergetic supply and intracellular network dynamics of mitochondria for the pathophysiological understanding of MDD. Mitochondria, organelles of mostly all eukaryotic cells, use carbon compounds to provide biochemical energy in terms of adenosine triphosphate (ATP). ATP is the bioenergetic currency and the main driver for enzymatic activity in all cells and tissues. Clinical symptoms of MDD including fatigue, difficulties concentrating, and lack of motivation were reported to be associated with impaired mitochondrial ATP production and changes in the density of the mitochondrial network. Additionally, the severity of these symptoms correlates negatively with mitochondrial functioning. Psychotherapy, antidepressant medication, and electroconvulsive therapy (ECT), a method used to treat severe and treatment-resistant forms of MDD, achieve robust antidepressant effects. The biological mechanisms beyond the treatment response to antidepressant strategies are partially understood. Here, mitochondrial functioning is discussed as a promising new biomarker for diagnosis and treatment effects in MDD.

## Introduction

With a lifetime prevalence of up to 20%, major depressive disorder (MDD) is one of the most common psychiatric diseases worldwide currently affecting 350 million people as estimated by the World Health Organization (WHO). Despite advances in etiological research and currently existing successful therapy strategies, MDD seems to be a steadily growing threat to the health systems. From a systemic point of view, MDD is not only associated with strong individual suffering and an increased suicidal probability but also with physical complications including cardiovascular^1^ and cerebrovascular^2^ impairments, hypertension^3^, diabetes^4^, autoimmune diseases^5^ and immunological impairments^4,6^, tumors and even cancer^7^. The relationship between MDD and higher risk factors for physical diseases is a well-documented finding in literature^8^. However, the causal (biomolecular) links have not been sufficiently identified. In sum, MDD is a serious and complex disease that affects not only the quality of the mental wellbeing of those affected but is also linked to the aforementioned complications that significantly increase morbidity and mortality^9^. This MDD-related somatic vulnerability points to an overall organic character, which might physiologically affect much more than just neurons in the brain. Considering other etiologic risk factors for MDD, negative life events, and stressors of sufficient duration, intensity, or frequency often precede depressive episodes^10^. These include traumatic life experiences^11^, e.g. loss of family members or individuals of comparable importance, but also psychosocial stressors^12^, such as loss of employment^13^, high psychosocial demands in social occupations^14^, nightshift work^15^, and the care of chronically ill relatives^16^. As another important factor in the etiology of MDD, adverse childhood experiences (ACE) including abuse and neglect, which negatively influence the stress resilience of affected individuals on the psychological as well as biological level, were identified. Chronic and traumatic stress in childhood is associated with an increased probability for the occurrence of MDD^17^, and lowers the threshold for higher symptom severity and causes a higher probability for a chronic course of the disorder^18,19^. One reason might lie in the fact that the responsiveness to antidepressant therapy might be psychobiologically altered by the consequences of ACE, including changes in the endocrine stress response signaling, immunological functioning as well as neurocognition (for a review see ref. ^20^). Therefore, a history of ACE needs to be considered with more attention in clinical treatment as well as in psychiatric research when evaluating clinical treatment responses and remission stability. Of particular pathophysiological significance is the reduction of sleep quantity and quality, which is a core symptom of various mental health conditions^21,22^. Healthy sleep is fundamental for the reconstitution of the bioenergetic resources of the body^23^. On the other hand, there are also protective factors that might increase the threshold level for developing MDD, which include positive psychosocial as well as lifestyle-related factors. For example, a high level of social integrity, as well as emotional support from partners, family, and friends, were identified as strong factors of resilience^24^. Stress coping strategies and mental stress management programs^25^, at best combined to relaxation techniques^26,27^ and sports also show protective effects in coping with everyday stressors, but also after chronic as well as traumatic life experiences. Research on the psychobiological stress-buffering effects of coping and mindfulness becomes an important and increasing new discipline in clinical MDD research.

## Oxidative stress and its role in the pathophysiology of MDD

The investigation as well as the characterization of the underlying pathophysiological mechanisms of MDD is of highest clinical interest: on the one hand to be able to diagnose MDD with the utmost certainty, and on the other hand for identifying and determining appropriate treatment approaches, e.g. based on robust and easily applicable biomarkers. Consequently, the demand for such markers (biomarkers or technical markers, i.e. markers ablated from electrophysiology or MRI-based neuroimaging) is correspondingly high, as they would allow the detectability of MDD (and also the subtype of it) with high sensitivity and specificity and open the gate for individual treatment strategies to make personalized medicine available in psychiatry. Contrary to the previous perspective that MDD is solely a brain disease, changes were described also on other biological levels outside the central nervous system (CNS). In addition to the changes in neurocognitive^28,29^, endocrine^30^, immunological^31^, and physiological functioning^32^, also biomolecular^33^, biochemical as well as bioenergetic alterations^34,35^ were reported in MDD. Under short-term conditions of stress, the physiological stress response is activated triggering a coordinated response from the body (somatic reaction). This causes a systemic activation of the central and peripheral nervous system, which in turn communicate with the endocrine system and the immune system to respond optimally to the stressor. In contrast, exposure to chronic or traumatic stress can lead to a maladaptive physiological condition that affects the functionality of the whole organism in the long term. This altered stress reactivity, which is considered to result from the biological dysregulation of the stress response orchestra, is accompanied by increased production rates of reactive oxygen species (ROS). To chemically activate inert molecular oxygen (O_2_), oxygen radicals are enzymatically generated as a side-product of oxidative phosphorylation (OXPHOS) inside mitochondria. Chronically increased ROS production rates are considered to be one core mechanism discussed in the etiology of MDD^36,37^, adding the main mediators for stress-associated biological damage in the body. At the biomolecular level, the sum of ROS negatively affects the antioxidant potential (AOP)—the sum of all antioxidants and their ROS-buffering capacity in cells and tissues. If the compensatory power of the AOP fails, a biochemical imbalance is created, which is defined as oxidative stress^38^. Based on their main production site, the mitochondria present in mostly all cells of the body, these ROS molecules non-specifically attack molecular cell structures as free radicals, including the mitochondria themselves, as well as cell membranes, proteins, and even the genome^36^. In addition to their role as major producers of ROS, mitochondria are the major source of biochemical energy in the form of adenosine triphosphate (ATP). ATP is the biochemical currency of all directed biological processes in eukaryotic cells, including immune cells. As a physiological consequence of ROS-associated stress, the soma develops a higher state of molecular chaos in terms of biological entropy. Damage to functional structures including the genome, the proteome, and other systemic functions of the cell might lead to a higher vulnerability for developing somatic as well as mental health conditions including MDD. To compensate for these biomolecular damages, cells have to invest energy in terms of ATP for biological processes like DNA repair, mitochondrial autophagy or the epigenetic regulation of gene expression to recover homeostasis by reversing ROS-induced entropy.

## Psychoneuroendocrine and immunological alterations in MDD

Psychoneuroimmunological research provided evidence that inflammatory processes play a key role in the manifestation and development of mental disorders. The first indications were based on observations that patients with MDD have a significantly increased body temperature^37^ as well as changes in the perception of pain^39^, which is accompanied by an increased pain sensitivity with simultaneously lowered pain threshold. One contributing mediator of these alterations is the C-reactive protein (CRP), which is mainly released by the liver in physiological conditions of systemic inflammation. Depressed patients show a discrete increase in serum CRP compared to non-depressed control subjects^40^. Also, immunological alterations occur in depressed patients, which are mainly based on changes in the composition of immune cell populations due to chronic stress^41^. On the other hand, immune cells from patients with MDD show reduced immunological reactivity^42^. These observations are in turn counteracted by increased levels of proinflammatory cytokines, including interleukin 1-beta, interleukin-6, and tumor necrosis factor-alpha (TNF-alpha). Despite the suppressed functionality of the immune system in MDD, the proinflammatory signaling molecules are elevated in blood serum^43^. The increased immunological cytokine production in MDD could also be demonstrated in in vitro experiments^43^. The precise immunoregulatory changes leading to these proinflammatory phenotypes in MDD are not completely identified, although mitochondria also seem to play a central role in this^44,45^.

## Mitochondrial bioenergetic activity as a new biomarker candidate in MDD

**Fig. 1:**
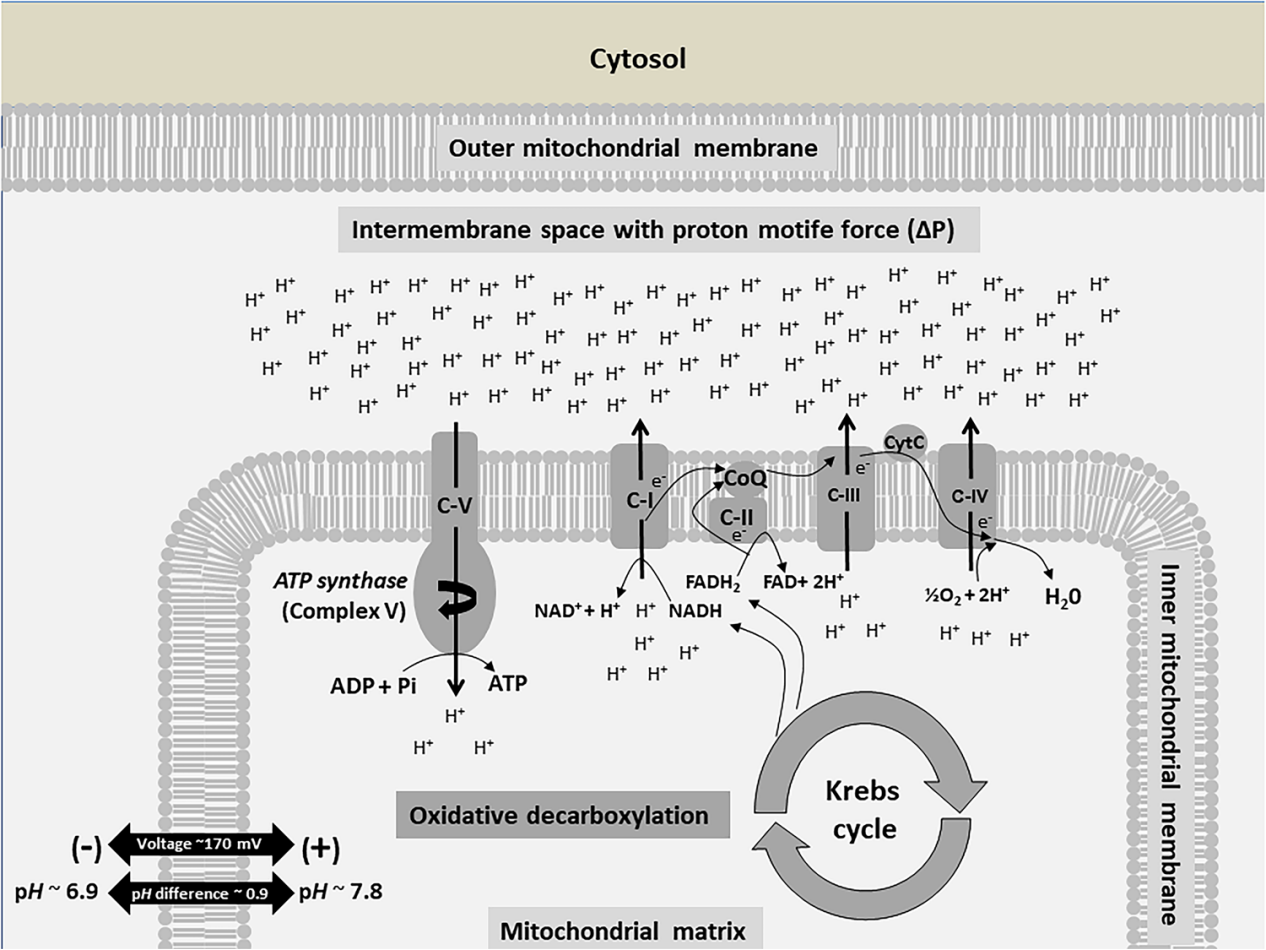
Schematic representation of the proton motive force (∆P) across the inner mitochondrial membrane to generate a proton gradient used to produce adenosine triphosphate (ATP) from adenosine diphosphate (ADP) and inorganic phosphate (Pi). Nicotinamide adenine dinucleotide (NAD) and Flavin adenine dinucleotide (FAD) possess redox capabilities to bind and to provide electrons (e^-^) as well as protons (H^+^). The electron transport chain consists of the complexes C-I - C-IV. Coenzyme Q (CoQ) and Cytochrome C (CytC) contribute to the electron transport chain as co-factors. Protons, electrons, and oxygen (O_2_) are used to generate water (H_2_O). Additionally, protons are shuttled into the intermembrane space to use ∆P for the generation of ATP at the transmembrane enzyme *ATP synthase* (Complex V).

The core metabolism of all eukaryotic cells is based on the possibility to chemically convert hydrocarbon compounds (e.g. sugars, lipids, and amino acids) into compounds of lower molecular weight by the enzymatic removal of carbon dioxide (CO_2_) to convert the free energy into biochemical energy in form of ATP. This process called *anaerobic respiration* is performed without the need for molecular oxygen (O_2_). The metabolic reactions of the anaerobic respiration are considered relatively inefficient and do not cover the complete energy demand of the cell. Phylogenetically derived from cyanobacteria, mitochondria are descendants of organisms that gave up their autonomy by endosymbiosis and became a compartment of the eukaryotic cell. They consist of an outer and an inner lipid bilayer. This particular anatomy allows mitochondria to function as a kind of biophysical battery, in which a charge separation based on protons (H^+^) is generated across the inner mitochondrial membrane. With sufficient charge, the resulting electrochemical potential leads to the proton motive force (PMF). Now, combined to an electron transport chain (ETC), consisting of proteins and co-factors integrated into the inner mitochondrial membrane, the PMF is used for the production of ATP together with the consumption of O_2_ (see Fig. 1 for a schematic representation of mitochondrial oxidative phosphorylation at the inner mitochondrial membrane).

**Fig. 2:**
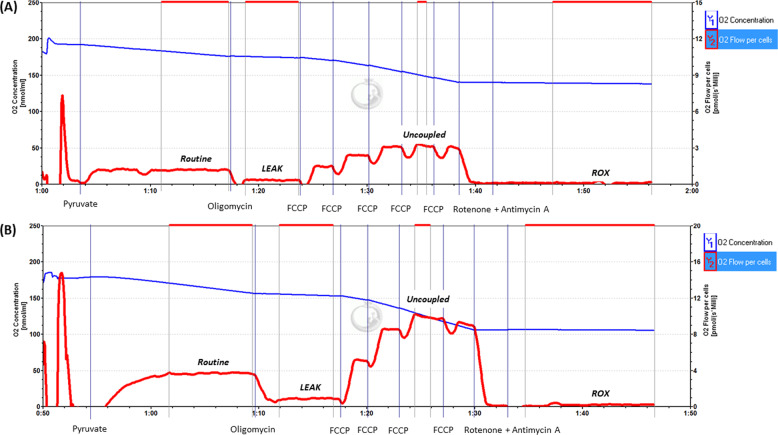
Representative high-resolution respirometry measurement to investigate mitochondrial bioenergetics in cells and tissues. Intact peripheral blood mononuclear cells (PBMC) were collected from whole blood of one depressed patient (a) and one sex- and age-matched, non-depressed control subject (b). Respirometric profiles of PBMC from depressed patients are characterized by less quantitative and qualitative respirometric activity compared to non-depressed controls. Respirometric characterization of peripheral blood immune cells is performed using high-resolution respirometry with O2K oxygraphs (Oroboros Instruments, Austria). Blue lines indicate oxygen concentration (nmol/ml) of the respirometry medium in air-sealed oxygraph chambers. Red lines show consumption rates of oxygen (pmol O_2_/s*10^6^ cells). To characterize the performance of mitochondrial respiration in intact PBMC a Substrate-Uncoupled-Inhibitor-Titration (SUIT) protocol is used to generate the following respirometric conditions: After adding the sample into the oxygraph chamber, the unstimulated cellular respiration level is measured (*Routine* respiration, R), followed by *Leak* respiration (L) after adding oligomycin, an *ATP-synthase* (Complex-V) inhibitor to the cells. The difference between *Routine* and *Leak* defines the respirometric activity under the production of ATP at C-V (*ATP-turnover related respiration*; R-L). Next, the mitochondrial system is manipulated with carbonilcyanide *p*-triflouromethoxyphenylhydrazone (FCCP), which depolarizes the membrane potential across the inner mitochondrial membrane. Titration of FCCP reveals the highest level of oxygen consumption (*Uncoupled* respiration). The difference between *Routine* and *Uncoupled* respiration determines the *Spare respiratory capacity*, the bioenergetics resources of the system to cover higher energy demands. *Residual oxygen consumption* (ROX) is the amount of background oxygen consumption independently from the electron transport system (ETS). It is induced by the blockage of Complex I with rotenone and Complex III with antimycin A, respectively. All respirometric parameters are corrected for ROX. For a detailed description of the procedure see Karabatsiakis et al.^35^.

Disorders of mitochondrial energy metabolism can be attributed to genetic diseases^46,47^ as well as to environmental stressors, including exposure to heavy metals, toxins, and other xenobiotic substances^48^. These bioenergetic impairments are usually harmful or even fatal^44^. Patients with mitochondriopathies show an increased risk for mental disorders, including MDD^45^. The causality of this observation could not be demonstrated yet. However, there are many indications that the biochemical correlates of biological energy production and their underlying mechanisms could be an explanatory approach for the loss of mental as well as somatic performance characteristics for patients with MDD. Initial studies on mitochondrial energy metabolism suggest that MDD is associated with an impaired bioenergetic supply and alteration of the intracellular mitochondrial network measured in immune cells collected from peripheral blood^49,50^. One first study demonstrated that the mitochondrial bioenergetic performance of peripheral blood mononuclear cells (PBMC) was significantly reduced in MDD. Additionally, the reduction of mitochondrial performance was significantly correlated with the severity of depressive symptoms reported by the patients^35^. These physiological changes may also be attributed to an adaptation of the mitochondrial network inside the cells, which seems to be sensitive to physiological as well as environmental stress^35^. In addition to immune cells, other blood components such as blood platelets show a significant reduction in their bioenergetic activity profile^34^. The expectation of mitochondrial involvement in the pathophysiology of MDD is furthermore supported by in -vitro findings based on cell culture research. These show that the mitochondrial energy metabolism of immune cells can be altered by exposure to selective serotonin reuptake inhibitors (SSRI^45^). Animal studies demonstrated that deletions in mitochondrial DNA (mtDNA) and resulting mitochondrial dysfunction in the *paraventricular thalamic nucleus* are associated with lethargic behavioral changes that are linked to emotional, vegetative, and psychomotor impairments^51^. These observed changes are likely to be transferable to humans as they are core symptoms of MDD. In sum, mitochondria and their bioenergetic functioning represent a new and innovative approach in translational research on MDD. The correlation between the mitochondrial bioenergetic activity and the severity of depressive symptoms^35^ raises the question of how the mitochondrial performance and the mitochondrial network re-align in the context of clinical recovery after antidepressant treatment towards healthy functioning. The mitochondrial bioenergetic profile (the function of bioenergetic activity and the intracellular load of mitochondria) derivable from blood samples could thus be used as a possible indicator of the energetic state of the patient (see Fig. 2 for one representative high-resolution respirometric activity measurement in PBMC of a depressed patient (A) and a non-depressed control subject (B)). This potential biomarker could help to measure relevant mental as well as physical impairments that result in fatigue, lack of energy, and difficulties concentrating, prominent symptoms in MDD^35^.

## Electroconvulsive therapy (ECT) and its efficacy in MDD

In addition to psychotherapy and psychopharmacology, ECT is also an effective intervention strategy. The clinical application of ECT goes back to work by Cerletti and Bini in the 1930s^52^. It is particularly used in the treatment of very severe forms or life-threatening psychiatric disorders including MDD. ECT is currently the most effective treatment option for severe, treatment-resistant MDD, and is also used in catatonia (and catatonic syndromes), rapid deterioration of the physical health of severely depressed patients, intolerance to antidepressant medication, and in MDD during pregnancy, amongst other conditions^53^. Additionally, it can be applied after the direct agreement of the patient to be treated with ECT, if former ECT treatments were effective^54,55^. At least half of the patients with treatment-resistant MDD show a significant improvement in the severity of depressive symptoms after ECT^56^. Clinical studies reported that ECT achieves significantly better outcomes compared to simulated ECT, placebo, and psychopharmacotherapy^57–59^. However, it is poorly understood which psychological and biological factors determine the response to ECT treatment. Nevertheless, the biological effects, as well as the mechanisms of action following ECT, remain postulated^58^ as their empirical evidence is still pending.

## ECT: an introduction to methodology and conduct

Under short-term anesthesia (only for few minutes without intubation) and after a subsequent application of a muscle relaxant (e.g. succinyl-choline), ECT induces an electric current through the brain via electrodes on the scalp, which leads to a targeted and synchronous depolarization of neuronal assemblies in the brain that results in a convulsive state. To achieve a therapeutically significant effect, the time interval of 20 seconds should not be deceeded in the current-induced convulsion according to the clinical guidelines^60,61^. In general, patients with MDD receive 9 to 12 sessions consecutive at intervals of up to 3 days^53,59,62^. In addition to the aforementioned predictors of treatment response, patients with a higher age^63,64^, patients with psychotic^64–66^ or “melancholic” depression^67^, and patients with a severe suicidal tendency^68,69^ respond particularly well to ECT. Another predictive factor for the treatment response to ECT is the rate of improvement in clinical symptom severity^58,70^, which can be observed after the initiation of therapy. Clinical studies reported that 83% of patients achieve full remission of depressive symptoms when they show a reduction in the severity of depressive symptoms by the sixth ECT treatment session^57^. On the other hand, worse response to ECT is seen in patients with a chronic history of MDD or a clinical relapse^60,71,72^. Here, also negatively associated dose-response effects are described^73,74^. The sex of the patient does not influence the response to ECT treatment. This finding is of special interest because the lifetime risk for MDD in women is two times higher than the risk in men. Accordingly, the proportion of women with MDD is correspondingly higher compared to men^75^. To the best of our knowledge, the impact of traumatic life events experienced in childhood, adolescence, and adulthood on the response to ECT has not been studied. This is surprising, as all three lifetime periods are associated with a higher prevalence of traumatic life events in women compared to men. Preliminary research has demonstrated that mitochondrial bioenergetic activity in PBMC collected from non-depressed women with ACE is increased. Additionally, this increase shows a dose-response relationship with the severity of ACE^73^ measured as the CTQ sum score that is used to retrospectively assess and determine maltreatment load^74^. The precise biochemical underpinnings of these observations and how they are linked to an increased risk to develop MDD after exposure to ACE need more research.

## Current explanatory models for the effects of ECT

In addition to the problem of missing biomarkers in clinical diagnostics in psychiatry, there are also no clinically-applicable biological indicators for the prediction of positive response to antidepressant treatment. Accordingly, it is of particular interest to investigate biomolecular and bioenergetic changes in effective MDD treatments to derive possible predictors. This approach also applies to a better understanding of the clinical response to ECT. Although “good” to “very good” antidepressant effects can be achieved on the individual level, these effects are not observed in all patients. So far, explanatory models for the mechanisms underlying ECT have been related to neuroendocrine^76^ and monoaminergic effects including changes in neurotransmitter levels of serotonin, dopamine, and norepinephrine^77^. Further research findings point to ECT effects at the level of neuroplasticity, the release of neurotrophic substances^78^, and the regeneration of existing brain tissue^79^. Functional magnetic resonance imaging (fMRI) in the CNS showed that patients have a significant difference in these factors before and after ECT^80^. Furthermore, it could be observed that the reduction of neuronal connectivity is associated with a reduction in depressive symptoms^81^. The precise biomolecular correlates of these ECT-inducible neuronal alterations remain unidentified. Neurobiological nuclear magnetic resonance (NMR) spectroscopy studies found that ECT treatment increases glutamine and glutamate levels in the brain which occur particularly in the anterior cingulate cortex (ACC)^82–84^ and lead to normalization. The ACC is of particular importance for altered cognitive error-perception processes (cognitive examination of executive errors and conflict monitoring) in psychiatric patients^85^, which are associated with suicidality, a major problem for severely depressed and psychotic patients. Studies indicate that ECT treatments also lead to biological changes outside the CNS^86–88^. An increase in norepinephrine in blood plasma after ECT was observed in non-melancholic, depressed patients^86^. Further studies show an effect of ECT on the levels of the dopamine metabolite homovanillic acid and 5-hydroxyindoleacetic acid (5-HIAA), a serotonin metabolite in cerebrospinal fluid^87^. A study by Okamoto and colleagues extended this finding to blood plasma^88^. Being present in the blood, these biomarkers allow an initial biochemical insight into the overall organic reactivity of the body to ECT treatment. Pioneering mass spectrometry studies on changes in lipid metabolism associated with mitochondrial energy metabolism in blood provided the first evidence that ECT also has effects on the physiological level in the periphery of the body^89–92^. Multivariate analysis of variance revealed that the levels of cholesterol and its subtypes HDL and LDL increase after treatment. The apolipoprotein A1 also showed an increased level after ECT in comparison to the unchanged apolipoprotein B^93^. These results confirmed initial findings on changes in cholesterol and its derivatives in mood disorders^88,89,93^. Changes in cholesterol levels as a function of depressive symptom relief after ECT treatment were also found in one first study by Ramamurthy and colleagues^90^. This is of interest in the context of mitochondrial biosynthesis, as mitochondria account for the majority of bioactive steroids synthesized from cholesterol and cholesterol metabolites^94,95^. Recent findings also indicate that O_2_ consumption in the CNS is associated with mitochondrial bioenergetic activity^94^. In theory, if the observation of altered O_2_ consumption in the periphery can be extended to the CNS, studies using signals based on blood oxygen-level dependency (BOLD) might be also influenced by differences in basal (baseline) oxygen consumption rates, further limiting MRI-based observations as long as differences in mitochondrial activity and/or density cannot be controlled in the statistical analyses. As a result, between-group differences in the BOLD-contrast might not be related to neuronal differences in response to a stimulus, but also as a consequence of differences in basal/baseline physiological O_2_ consumption rates. Therefore it can be expected that mitochondrial respiratory activity will receive increasing scientific attention in the future. Taking into account the fact that all forms of physiological activity—in particular, enzymatic performance - require biochemical energy in form of ATP, recent observations on immunological effects by ECT are also interesting. Although the data is very limited first investigations also show effects of ECT at the immunological level^95^. The dependency of the immunocellular activity on the biochemical energy supply by mitochondria has not been investigated yet. However, this assumption and initial confirmatory data indicate that the bioenergetic activity of cells and tissues can provide a new, important factor in the understanding of (patho)physiological changes in MDD.

## Mitochondrial energy metabolism as an innovative candidate to study the ECT mechanism of action

Transdisciplinary trauma research failed to identify an applicable biological predictor for the individual response of depressed patients to ECT treatment. It is reasonable to believe that this aim is only intensified by the consideration of both clinical and biological factors^6^. Modern psychiatry also targets this goal in terms of personalized medicine. Studies will show to what extent the effects of ECT can be observed not only in the CNS but also on mitochondrial performance profiles measured in blood samples to be used as potential biomarkers. It is conceivable that the application of electric current during ECT also results in an electric current in the whole body which leads to a charge transfer that directly or indirectly affects the PMF inside the mitochondria. Another hypothesis involves a possible adaptation of the mitochondrial performance profile (respiratory activity and the intracellular load of mitochondria) by ECT. The characterization of ECT effects at the level of mitochondrial bioenergetic activity will provide important insights into the biological understanding of MDD. From a holistic perspective, the interdisciplinary combination of psychological, psychiatric, immunological, physiological, and biomolecular methods can be used to provide a much more systemic view on MDD. Secondly, significantly more knowledge about the biological effects of ECT on the severity of depressive symptoms and the response and progression of the ECT treatment could be gained. Inter- and transdisciplinary research approaches can help to boost the current knowledge about the biochemical and biological underpinnings of psychiatric disorders and the treatment options to catalyze the improvement of clinical therapeutic options and recovery rates. In addition to improved treatment strategies, the observed changes could also be used to derive potential new therapeutic methods that specifically target the mitochondrial bioenergetic activity. An appropriate starting point would be the field of orthomolecular medicine, which focuses on the influence of nutrition on the overall health of the organism. In the context of gene x environment interactions, nutrition shows a strong influence on biological systems including inflammation^96^, free-radical production, and oxidative stress^38^, DNA damage^97^, and most probably also on mitochondrial functioning^98^. In sum, deriving bioenergetic profiles from blood immune cells in stress and trauma-related diseases is a completely new discipline (suggested field: “*Experimental Bioenergetics in Clinical Psychology and Psychiatry”*) and is considered to be a very promising new approach to the development of a biological marker of mental illness and its therapeutic course. Accordingly, it is now necessary to initiate translational research in which these possible functional improvements can be objectified by interdisciplinary research methods. Mitochondrial energy metabolism is an innovative aspect in this regard. If ECT effects occur on the level of ATP production and O_2_ consumption, blood samples used to characterize mitochondrial performance in immune cells would be an easily implemented and well-tolerated approach that provides information about the bioenergetic state and thus potentially the somatic functionality of the patient. This would make the goal of personalized medicine in modern psychiatry become significantly more feasible. Another task of modern psychiatry must be to also consider environmental and lifestyle factors much stronger than today. For example, ACE and other types of critical life events combined with the available knowledge about biological vulnerability markers for stress susceptibility (genetic risk factors) should be taken into account in the sense of (gene)^n^ x environment interactions to significantly improve psychiatric care and its efficiency. This new perspective is also necessary and even today applicable to the context of ECT.
