# Correction: Depression, mitochondrial bioenergetics, and electroconvulsive therapy: a new approach towards personalized medicine in psychiatric treatment - a short review and current perspective

**DOI:** 10.1038/s41398-020-00973-5

**Published:** 2020-08-10

**Authors:** Alexander Karabatsiakis, Carlos Schönfeldt-Lecuona

**Affiliations:** 1 Institute of Psychology, University of Innsbruck, Innsbruck, Tyrol Austria; 2 Clinic for Psychiatry and Psychotherapy III, Ulm University Clinic, Ulm, Baden-Wuerttemberg, Germany

Correction to: *Translational Psychiatry*

10.1038/s41398-020-00901-7 published online 9 July 2020

In the original Article, Dr. Karabatsiakis and Prof. Dr. Schönfeldt-Lecuona’s names were indexed incorrectly as our system registered their academic titles as part of their names. We have corrected their names in the XML (this change will reflect in Pubmed).

